# Recent advances in the self-referencing embedded strings (SELFIES) library

**DOI:** 10.1039/d3dd00044c

**Published:** 2023-07-01

**Authors:** Alston Lo, Robert Pollice, AkshatKumar Nigam, Andrew D. White, Mario Krenn, Alán Aspuru-Guzik

**Affiliations:** a Department of Computer Science, University of Toronto Canada alston.lo@mail.utoronto.ca r.pollice@rug.nl alan@aspuru.com; b Chemical Physics Theory Group, Department of Chemistry, University of Toronto Canada; c Stratingh Institute for Chemistry, University of Groningen The Netherlands; d Department of Computer Science, Stanford University California USA; e Department of Chemical Engineering, University of Rochester USA; f Max Planck Institute for the Science of Light (MPL) Erlangen Germany; g Vector Institute for Artificial Intelligence Toronto Canada; h Canadian Institute for Advanced Research (CIFAR) Lebovic Fellow Toronto Canada

## Abstract

String-based molecular representations play a crucial role in cheminformatics applications, and with the growing success of deep learning in chemistry, have been readily adopted into machine learning pipelines. However, traditional string-based representations such as SMILES are often prone to syntactic and semantic errors when produced by generative models. To address these problems, a novel representation, SELF-referencing embedded strings (SELFIES), was proposed that is inherently 100% robust, alongside an accompanying open-source implementation called selfies. Since then, we have generalized SELFIES to support a wider range of molecules and semantic constraints, and streamlined its underlying grammar. We have implemented this updated representation in subsequent versions of selfies, where we have also made major advances with respect to design, efficiency, and supported features. Hence, we present the current status of selfies (version 2.1.1) in this manuscript. Our library, selfies, is available at GitHub (https://github.com/aspuru-guzik-group/selfies).

## Abstract 2

We describe the current state of the SELFIES library (version 2.1.1), and, in particular, the advances and improvements we have made in its underlying algorithms, design, and API.

## Graphical abstract

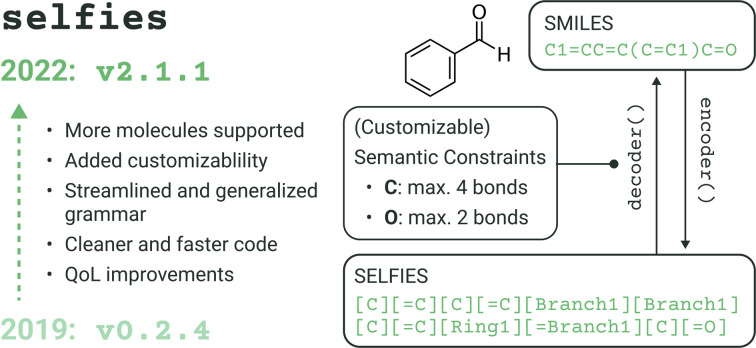

## 1. Introduction

In recent years, machine learning (ML) has become a powerful tool to tackle challenging problems in chemistry. Machine learning pipelines involve three crucial elements: data, representations, and models. Choosing the proper representation is important as it defines the space of models available to work with the data, as well as impacting directly model performance. For molecules, one of the more widely-used classes of representations encode molecules as strings (*i.e.*, the string-based molecular representations). These representations are popular since they can leverage the rich collection of ML tools that have been developed for sequential data.^1,2^ Historically, the most employed string representation is the Simplified Molecular Input Line Entry System (SMILES), which was introduced by Weininger in 1988.^3^ Currently, SMILES has become the *de facto* standard representation in cheminformatics and has historically been a key component of central applications in the field, such as chemical databases. The main appeal of SMILES is its simple underlying grammar, which allows for the rigorous specification of molecules in a manner that can be parsed efficiently, and which is readable for humans at least for small molecules.

However, in an ML setting, this grammar can carry two intrinsic weaknesses. First, many strings constructed from SMILES symbols are syntactically invalid due to the rigidity of the SMILES grammar, *i.e.*, the strings cannot be interpreted as molecular graphs.^4,5^ In particular, SMILES requires branch brackets and ring numbers to appear in matching pairs (*e.g.*, C(CC and C1C are invalid), so a single misplaced or missing token could ruin the validity of a SMILES string. This is problematic because ML models that produce SMILES strings, especially generative models, can be prone to these syntactic errors, rendering a significant fraction of their output meaningless. One strategy is to constrain the ML architecture to reduce the number of invalid structures, which has been demonstrated successfully in the literature.^6–8^ This approach, of course, needs significant computational effort and cannot be transferred directly to other systems without model retraining, model architecture adjustments, or domain-specific design considerations. An alternative and more fundamental solution is to define representations that are inherently robust. A first step towards this direction was taken by DeepSMILES,^9^ a string-based representation derived from SMILES that reworked some of its most syntactically susceptible rules. While DeepSMILES solves most of the syntactical errors, it does not address the second weakness of SMILES, namely, that even syntactically valid strings may not necessarily correspond to a physical molecule. Typically, this occurs when a string represents a molecular graph that exceeds normal chemical valences, in which case we call the string semantically invalid. For example, the SMILES string CO=C is semantically invalid because it erroneously specifies a trivalent oxygen atom, which is chemically unstable and reactive.

To eliminate both syntactic and semantic invalidities in string-based molecular representations on a fundamental level, an entirely new representation termed SELF-referencIng Embedded Strings (SELFIES) has been proposed by some of us.^10^ By construction, SELFIES is 100% robust to both syntactic and semantic errors. That is, any combination of SELFIES symbols specifies a molecular graph that obeys chemical valences. This is achieved through a small Chomsky type-2, context-free grammar^11^ that is augmented with self-referencing functions to handle the generation of branches and rings. Since its release, SELFIES has enabled or improved numerous applications, ranging from molecular design^12–15^ to interpretability^16^ to image-to-string and string-to-string translations,^17,18^ and has been extended to incorporate functional groups and other fragments.^19^ For an extensive summary of its applications and opportunities, we refer readers to the recent community paper on SELFIES.^20^

Herein, we introduce selfies 2.1.1, the latest version of the open-source Python implementation of SELFIES. In particular, we provide a detailed look into its history, developments, underlying algorithms, design, and performance. Together with the community, we have recently overviewed potential extensions and formulated 16 concrete future projects for SELFIES and other robust molecular string representations.^20^ We hope that this manuscript will also help in developing some of these extensions and ideas. Our software package selfies can be installed with “pip install selfies” and is available at GitHub (https://github.com/aspuru-guzik-group/selfies) under the Apache 2.0 license, along with comprehensive documentation and tutorials.

## 2. Timeline and advances

The selfies library version that implemented the representation from Krenn *et al.*^10^ was first released as selfies 0.2.4 in 2019. This older version provided an API of two translation functions where a restricted subset of organic, uncharged, nonaromatic SMILES strings could be converted to and from SELFIES strings. In addition, the internal algorithms behind selfies relied heavily on direct string manipulations, so they were computationally inefficient and difficult to maintain. Since then, selfies has undergone several major redesigns that have significantly advanced the algorithmic handling of both SMILES and SELFIES. Most importantly, the underlying grammar of selfies has been streamlined and generalized in subsequent versions. We will now describe the changes up until selfies 2.1.1, the most recent version of selfies at the time of publication of this work.

One major modification we made is that selfies now uses directed molecular graphs to internally represent SMILES and SELFIES strings. This has afforded selfies greater efficiency and flexibility, and enabled a number of additional extensions to be made. For example, we added support for aromatic molecules by kekulizing SMILES strings with aromatic symbols before they are translated into SELFIES. Furthermore, we handle species with partial charges, radicals, explicit hydrogens, non-standard isotopes, and stereochemical definitions in a fully syntactically and semantically robust way. Besides the standard constraints for the number of valences, users can now specify their own constraints and we provide built-in relaxed and stricter constraint presets that can be selected conveniently. Most recently, we introduced the ability to trace the connection between input and output tokens when translating between SELFIES and SMILES. Table 1 gives a brief changelog of the major releases of selfies and their associated advancements.

**Table 1:** A timeline of the various releases of selfies

Version	Year(s)	Description
0.1.1	(Jun) 2019	• Initial release of selfies
0.2.4	(Oct) 2019	• Release of selfies that implements the representation from Krenn *et al.*^10^
1.0.x	2020–21	• Expanded the support of selfies to a greater subset of SMILES strings, including strings with aromatic atoms, isotopes, charged species, and certain stereochemical specifications. To do so, the underlying grammar used by selfies was both streamlined and generalized
		• Added support for the customization of the semantic constraints used by selfies
		• Significantly improved the efficiency of translation between SELFIES and SMILES
		• Added a variety of utility functions to make the handling of SELFIES strings convenient
2.0.x	2021	• Updated the SELFIES alphabet to be more human-readable and standardized
		• Improved handling of stereochemical specifications in SELFIES involving ring bonds
2.1.x	2022	• Added support for explaining translations between SELFIES and SMILES through attributions

While the ideas outlined in the initial publication^10^ that ensure the validity of the representation remain at the core of selfies, the manifold implementation improvements and extensions are the novelties that we detail in this paper. Hereafter, unless specified otherwise, we will use selfies to refer to selfies 2.1.1 in particular and SELFIES to refer to the representation that selfies 2.1.1 implements. We will provide a complete and formal description of the updated representation in Section 3 and describe the API of selfies in Section 4.

## 3. SELFIES specification

Being 100% robust, every string of SELFIES symbols corresponds to a SMILES string that is both syntactically and semantically valid. Recall that we call a SMILES string semantically valid if it is syntactically valid and represents a molecular graph that obeys normal chemical valences.

Within SELFIES, these chemical valences are encoded as a constraint function 
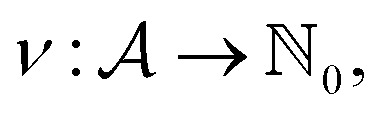
 where 
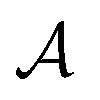
 is a finite universe of the atom types (*e.g.*, 
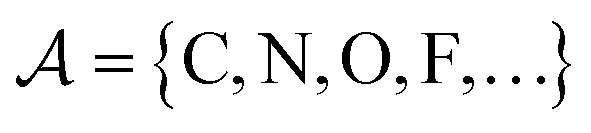
) of interest and 
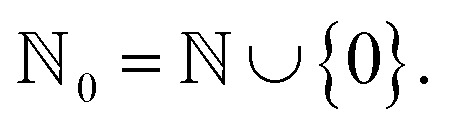
 The valences represented by *ν* dictate that an atom *A* must assume *ν*(type(*A*)) incident bonds in total. Note that if a SMILES string obeys the valences *k*, each of its atoms *A* makes at most *ν*(type(*A*)) explicit bonds within the string. There is a possibly-strict inequality in this case due to the way SMILES automatically adds implicit hydrogens until chemical valences are satisfied. In practice, the mapping *ν* is rationally chosen to align with both physical considerations and established cheminformatics packages such as RDKit.^21^ For example, a plausible setting might map

1 *ν*(C) = 4, *ν*(N) = 3, *ν*(O) = 2, *ν*(F) = 1

which is the default behaviour of selfies (see Section 4.3).

We formulate chemical valences in this manner to emphasize that although SELFIES depends on *ν*, it is not fixed to any particular setting of *ν*. That is to say, SELFIES can enforce rule sets induced by any arbitrary mapping 
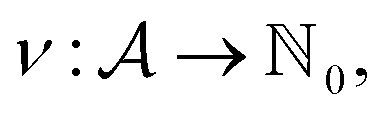
 even if they are not chemically meaningful. To highlight an absurd example, the uniform constraints *ν*(·) = 1000 can be used in principle, which corresponds to effectively having no semantic constraints at all. In this sense, SELFIES can be thought of as a general framework for an adjustable set of constraints *ν*. In the ensuing discussion, we will describe SELFIES under the assumption that some constraint function *ν* is fixed beforehand.

### 3.1. Syntax

Before explaining the SELFIES specification, we make a brief aside and give an overview of the form of SELFIES strings. Simply, a valid SELFIES string is any finite sequence of SELFIES symbols joined together. For ease of visual partitioning, all SELFIES symbols are enclosed by square brackets. Hence, a generic SELFIES string is of the form

2 […][…][…]⋯[…][…]

where the … is a placeholder for a symbol-specific token. We can further categorize SELFIES symbols into four main types, namely, atom, ring, branch, and miscellaneous, and characterize the syntax of each in the following. Throughout, let *ε* be the empty string and given *n* strings 
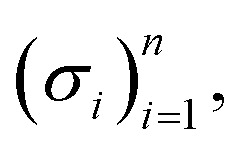
 let *σ*_1,_*σ*_2_,…,*σ*_*n*_ denote their concatenation.

#### 3.1.1. Atom symbols

The general SELFIES atom symbol has the form3
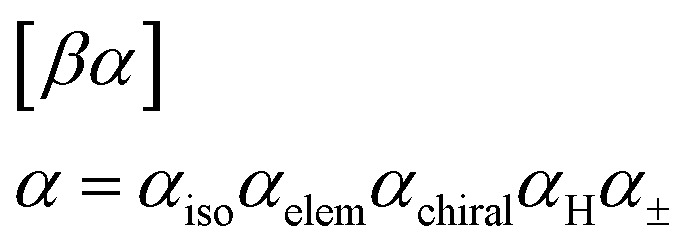
where *β* ∈ {*ε*, = , #,/, \} is a SMILES-like bond symbol and4
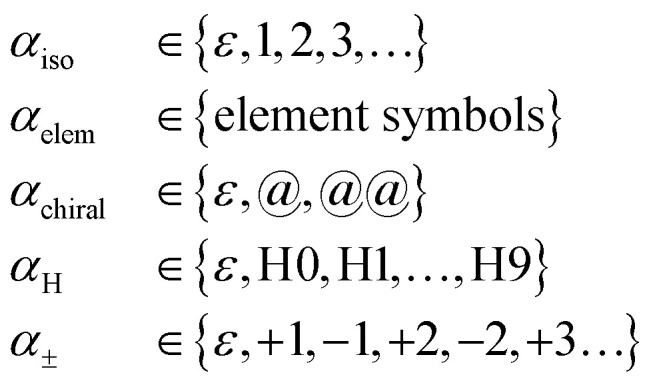


collectively specify an atom type type(*α*) in a SMILES-like fashion (the atom's isotope number, atomic number, chirality, number of attached hydrogens, and charge, respectively, and sometimes optionally). Notably, each SELFIES atom symbol is semantically unique, *i.e.*, different atom symbols are not interchangeable. This is not the case in SMILES due to shorthand abbreviations in how attached hydrogens and charge can be represented. For example, the SMILES atom symbol pairs ([Fe++], [Fe+2]) and ([CH], [CH1]) are interchangeable. To create a more standardized alphabet of symbols, we remove this redundancy in SELFIES.

#### 3.1.2. Branch symbols

The general SELFIES branch symbol has the form

5 [*β*Branch*

<svg xmlns="http://www.w3.org/2000/svg" version="1.0" width="13.454545pt" height="16.000000pt" viewBox="0 0 13.454545 16.000000" preserveAspectRatio="xMidYMid meet"><metadata>
Created by potrace 1.16, written by Peter Selinger 2001-2019
</metadata><g transform="translate(1.000000,15.000000) scale(0.015909,-0.015909)" fill="currentColor" stroke="none"><path d="M480 840 l0 -40 -40 0 -40 0 0 -40 0 -40 -40 0 -40 0 0 -120 0 -120 -80 0 -80 0 0 -40 0 -40 40 0 40 0 0 -80 0 -80 -40 0 -40 0 0 -80 0 -80 40 0 40 0 0 -40 0 -40 80 0 80 0 0 40 0 40 40 0 40 0 0 40 0 40 -40 0 -40 0 0 -40 0 -40 -40 0 -40 0 0 160 0 160 40 0 40 0 0 40 0 40 40 0 40 0 0 40 0 40 40 0 40 0 0 40 0 40 40 0 40 0 0 80 0 80 -40 0 -40 0 0 40 0 40 -40 0 -40 0 0 -40z m80 -120 l0 -80 -40 0 -40 0 0 -40 0 -40 -40 0 -40 0 0 80 0 80 40 0 40 0 0 40 0 40 40 0 40 0 0 -80z"/></g></svg>

*]

where *β* ∈ {*ε*, = , #} is a SMILES-like bond symbol and *ℓ* ∈ {1, 2, 3}.

#### 3.1.3. Ring symbols

SELFIES ring symbols can be further subdivided into two sub-types. These are of the form6
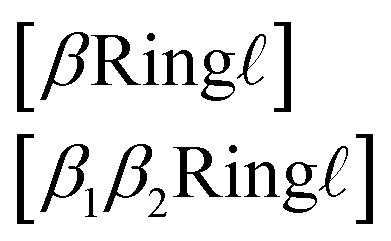
where *β* ∈ {*ε*, = , #} and

7 *β*_1_,*β*_2_ ∈ {−,/,\}, and not both *β*_1_ = *β*_2_ = −

are SMILES-like bond symbols and *ℓ* ∈ {1, 2, 3}, similar to branch symbols. The second ring symbol type (eqn (6)) is used to handle stereochemical specifications across ring bonds (see Section 3.5).

#### 3.1.4. Miscellaneous symbols

SELFIES has a few auxiliary symbols that are not core to the representation. These symbols still have common use cases and are specially recognized by the functions in selfies that translate between SELFIES strings and SMILES strings (see Section 4.1):

• The dot symbol, which can be used to express multiple disconnected fragments in a single SELFIES string, similar to its role in SMILES. The dot symbol is interpreted by treating it as delimiter and splitting the SELFIES string across the symbol. Then, each token is treated as an independent SELFIES string.

• The [nop] (for “no-operation”) symbol, which is a special padding symbol ignored by selfies.

Table 2 provides examples of SELFIES atom, branch, and ring symbols.

**Table 2:** Example SELFIES symbols, by symbol type

Type	Examples
Atom	[#13C], [=O], [C@@H1], [N + 1]
Branch	[Branch3], [#Branch1], [=Branch2]
Ring	[=Ring1], [/\Ring3], [Ring2]
Misc.	., [nop]

### 3.2. The SELFIES grammar

Now, we return to explaining the practical algorithm used to derive SMILES strings from their corresponding SELFIES strings. To do so, we first introduce the notion of a context-free grammar. A context-free grammar *G* is a tuple *G* = (*V*, *Σ*, *R*, *S*), where *V* and *Σ* are disjoint finite sets of nonterminal and terminal symbols, respectively, *R* ⊆ *V* × (*V* ∪ *Σ*)* is a finite relation,††The Kleene star of a finite set of symbols *A*, denoted A*, is the set of all strings formed by concatenating finitely-many symbols from *A*, which includes the empty string. and *S* ∈ *V* is a so-called start symbol. Under *G*, strings of terminal symbols can be derived by performing a finite sequence of replacements starting with the single-symbol string *σ*_0_ = *S*. At each step *t*, if the current string *σ*_*t*_ contains a nonterminal symbol ***A*** ∈ *V* (*i.e.*, *σ*_*t*_ = *ρ*_1_***A****ρ*_2_ for *ρ*_1_, *ρ*_2_ ∈ (*V* ∪ *Σ*)*) and there is an (***A***, *α*) ∈ *R*, then we replace ***A*** with *α* to get the next string *σ*_*t*+1_ = *ρ*_1_*αρ*_2_. For this reason, tuples (***A***, *α*) ∈ *R* are called production rules, and are suggestively notated ***A*** → *α*. The derivation terminates once only terminal symbols remain. The derivation of SMILES strings under SELFIES is similar to the preceding process. In fact, a context-free grammar underlies SELFIES, which we call the SELFIES grammar.

Specifically, the SELFIES grammar takes8
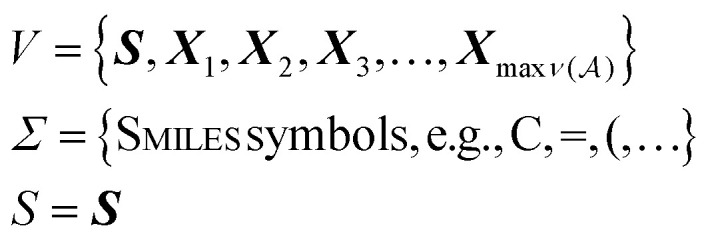
where 
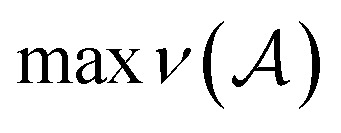
 is the maximum valence of all atom types. The production rules *R* will be characterized later. Given a SELFIES string, its corresponding SMILES string is then derived through a trajectory of replacements starting from *S*, as previously described. However, there are two further modifications that provides SELFIES its strong robustness. First, the replacements that are performed are not chosen arbitrarily, but are instead dictated by the SELFIES string of interest. At each derivation step, the next symbol of the SELFIES string is read off and fully specifies which production rule is applied. We systematically design this symbol-to-rule mapping such that the final derived SMILES string will always be valid. Second, SELFIES augments the grammar with self-referencing functions. These self-referencing functions manipulate the derivation process in more complicated ways than simple replacements, so they are not production rules. However, as before, the manner in which these self-referencing functions are applied is also dictated by the symbols in the SELFIES string. Thus, a SELFIES string can be viewed as a recipe of instructions (the symbols) that guides string derivation under the SELFIES grammar.

### 3.3. Simple chain derivation

Herein, we begin by considering the simplest type of SELFIES strings, those that correspond to simple chains of atoms. In SMILES, simple chains of atoms are represented by sequences of alternating atom and bond SMILES symbols, the latter of which can sometimes be left implicit by convention. Examples of such SMILES strings include CCCC (*n*-butane) and O=C=O (carbon dioxide). Analogously, in SELFIES, simple chains are represented by sequences of SELFIES atom symbols, which can be understood as playing a similar role as a grouping of a SMILES atom symbol and its preceding SMILES bond symbol. Simple chains are the easiest to derive in SELFIES, because the process occurs only through mere replacements, as in regular context-free grammars.

The derivation of a simple chain starts with the initial string *σ*_0_ = *S*. Recall that the SELFIES symbols dictate how production rules are applied. For simple chains, this is achieved by having each pair of SELFIES atom symbol and nonterminal symbol *A* ∈ *V* determine a production rule of the form *A* → *α A*′, where *α* ∈ Σ* is a terminal string and *A*′ ∈ *V* ∪ {*ε*}. Then, a sequence of replacements is iteratively performed by treating the SELFIES string as a queue 
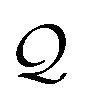
 of SELFIES symbols. At each step, the head of 
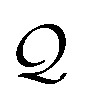
 is popped‡‡To pop or dequeue the head of a queue 
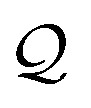
 means to fetch and then remove the oldest item in 
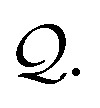
 and, with a nonterminal symbol in the current string *σ*_*t*_, is used to select and apply a production rule to get the next string *σ*_*t*+1_. Note that *σ*_0_ = *S* is itself a single nonterminal symbol, and each rule induced by a SELFIES atom symbol replaces one nonterminal symbol by another. Hence, throughout the derivation, the current string *σ*_*t*_ will always contain at most one nonterminal symbol and there is never any ambiguity as to how or which production rule is applied. Once the current string has only terminal symbols or 
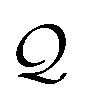
 is empty, the process ends (since SELFIES strings are finite, termination necessarily occurs). The final derived SMILES string is read off by dropping all nonterminal symbols.

We now fully enumerate the SELFIES atom symbol to production rule mapping. Let [*βα*] be a generic atom symbol, as described in eqn (3). Based on this symbol, we first define the terminal string9
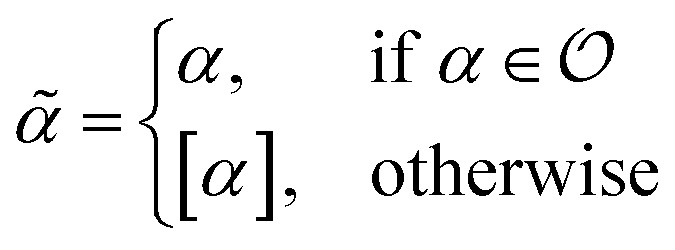
where 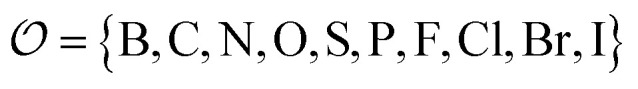 are the symbols of elements in the SMILES organic subset. The string 
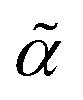
 can be thought of as transforming *α* into the SMILES syntax. Then [*βα*] together with the nonterminal symbol *S* ∈ *V* specifies the production rule:

10 ***S*** → *

<svg xmlns="http://www.w3.org/2000/svg" version="1.0" width="14.727273pt" height="16.000000pt" viewBox="0 0 14.727273 16.000000" preserveAspectRatio="xMidYMid meet"><metadata>
Created by potrace 1.16, written by Peter Selinger 2001-2019
</metadata><g transform="translate(1.000000,15.000000) scale(0.015909,-0.015909)" fill="currentColor" stroke="none"><path d="M240 720 l0 -80 40 0 40 0 0 40 0 40 80 0 80 0 0 -40 0 -40 120 0 120 0 0 80 0 80 -40 0 -40 0 0 -40 0 -40 -80 0 -80 0 0 40 0 40 -120 0 -120 0 0 -80z M240 520 l0 -40 -40 0 -40 0 0 -80 0 -80 -40 0 -40 0 0 -120 0 -120 40 0 40 0 0 -40 0 -40 80 0 80 0 0 40 0 40 40 0 40 0 0 40 0 40 40 0 40 0 0 -80 0 -80 80 0 80 0 0 40 0 40 40 0 40 0 0 40 0 40 -40 0 -40 0 0 -40 0 -40 -40 0 -40 0 0 160 0 160 40 0 40 0 0 80 0 80 -40 0 -40 0 0 -40 0 -40 -40 0 -40 0 0 40 0 40 -120 0 -120 0 0 -40z m240 -160 l0 -120 -40 0 -40 0 0 -40 0 -40 -40 0 -40 0 0 -40 0 -40 -80 0 -80 0 0 120 0 120 40 0 40 0 0 80 0 80 120 0 120 0 0 -120z"/></g></svg>

****X***_**_

where ** = *ν*(type(*α*)) is the valence of the atom type specified by *α*, and we hereafter define *X*_0_ = *ε* to be the empty string to handle the case where *ℓ* = 0. The atom symbol [*βα*] together with the symbol *X*_*i*_ ∈ *V*, where 
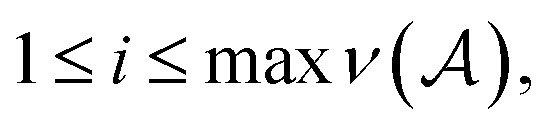
 specifies a production of the form:11
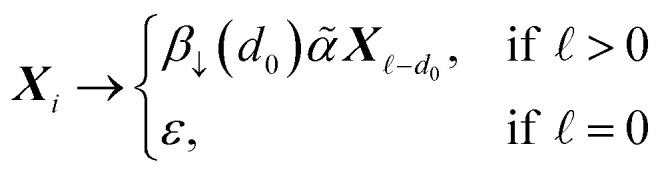
where *d*_0_ = min(*ℓ*, *i*, *d*(*β*)). Here, *d*(*β*) is a function that returns the order of the bond type represented by *β*:12
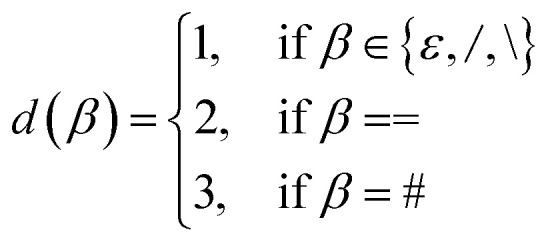
and *β*_↓_(*n*) is a function that demotes *β* into a SMILES token representing a bond of lower order *n* ≤ *d*(*β*):13
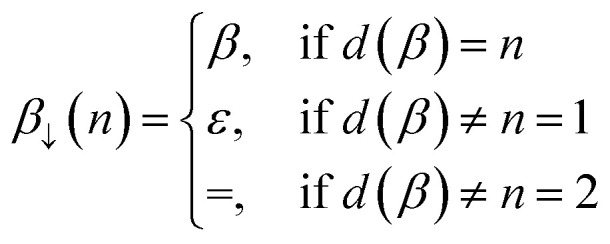


In eqn (10) and (11), the nonterminal symbols *X*_m_ are intuitively memorizing the maximum number of bonds that the most recently derived atom can adopt; the nonterminal symbol *X*_m_ can be understood as encoding that the last atom can make at most m bonds. When the next atom is derived, the bond connecting it to the preceding atom has its order decreased minimally such that the bond constraints are always satisfied.

Examples: To show these production rules in a concrete setting, we will translate the SELFIES string14
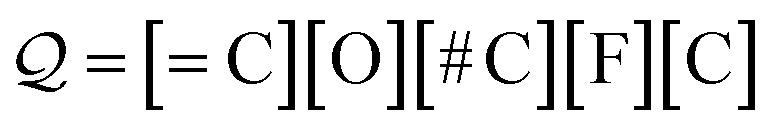


along with the constraints in eqn (1). The derivation of its corresponding SMILES string would proceed step-wise as follows:15
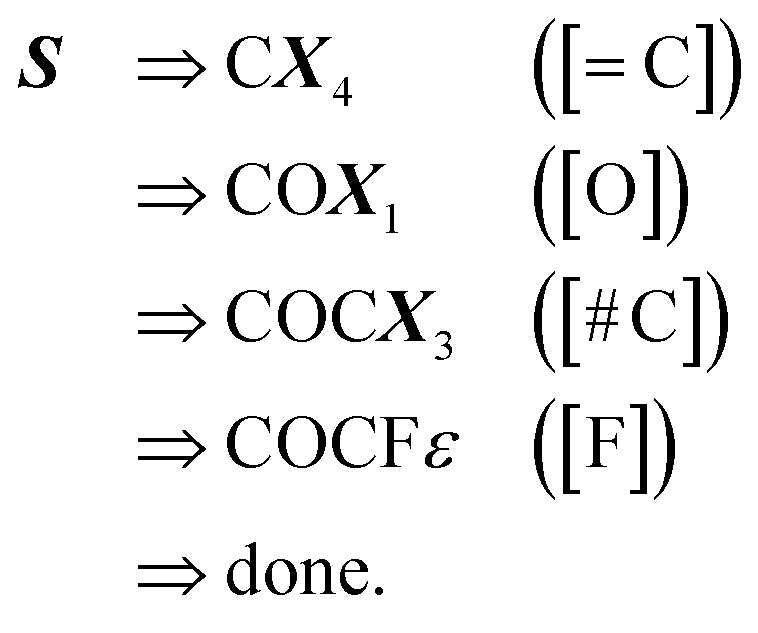
where each line *σ*_*t*_ ⇒ *σ*_*t*+1_ ([*βα*]) is used to denote a step of the derivation process induced by the SELFIES symbol [*βα*]. The final derived SMILES string in this case is COCF. Now, for a more complicated example, consider the SELFIES string16
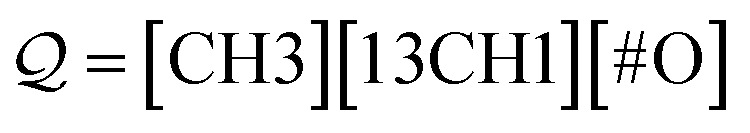


under the same constraints. The derivation proceeds as17
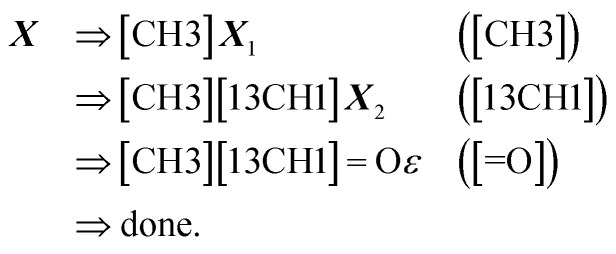


producing the final SMILES string [CH3][13CH1]=O. Note that isotopes are assumed to share the same valence, and when hydrogen atoms are specified in an atom type, its valence is decremented accordingly.

### 3.4. Branch derivation

So far, we discussed chains of atoms, and their connectivity. However, most molecules are more complex than simple linear chains. Therefore, now, we talk about the derivations of branches (followed by rings in the subsequent section). In SMILES, branches are specified by enclosing a SMILES substring in parentheses, which can be recursively nested; for example, CC(=O)O (acetic acid) and C(=O)(C(=O)O)O (oxalic acid). In SELFIES, branches are specified by SELFIES branch symbols, and similar to atom symbols, every pair of SELFIES branch symbol and nonterminal symbol determine some rule on how to modify the current string. We can encode branched trees of atoms in SELFIES by sequences of atom and branch symbols.

The derivation process extends that for simple chains (in Section 3.3), where we pop SELFIES symbols step-by-step off of a queue 
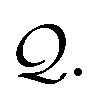
. We only add an additional rule for when we dequeue a branch symbol from 
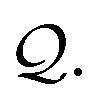
. Let this symbol be [*β*Branch*ℓ*], as in eqn (5), and let *A* be a nonterminal symbol in the current string *σ*_*t*_. If *A* ∈ {*S*, *X*_1_}, then this specifies the application of the production rule *A* → *A*. Effectively, the branch symbol is ignored in this case. If *A* = *X*_*i*_ for *i* ≥ 2, then we perform a replacement:

18 ***A*** → *ρ****X***_*i*−d_0__

where *d*_0_ = min(*i* − 1, *d*(*β*)), and *ρ* ∈ Σ* is a SMILES substring obtained through the following recursive process.

First, *ℓ* symbols are popped from 
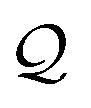
 and converted into integer values by the mapping summarized in Table 3. Let *c*_1_⋯, *c*_*ℓ*_ be the indices in first-to-last order of retrieval. In the event that 
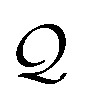
 contains fewer than *ℓ* symbols, the missing indices are set to have a default value of 0. Next, these indices are identified with a natural number 
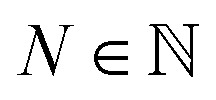
 by treating them as hexadecimal digits:19
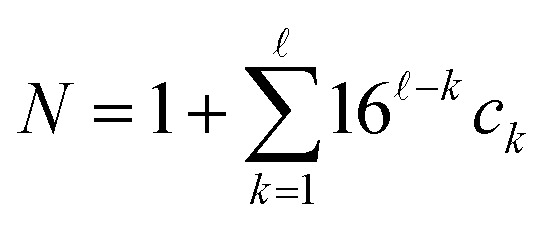


**Table 3:** The symbols succeeding a branch or ring SELFIES symbol are sometimes overloaded with a numeric index, which is determined by the following symbol-to-index mapping

Index	Symbol	Index	Symbol
0	[C]	8	[#Branch2]
1	[Ring1]	9	[O]
2	[Ring2]	10	[N]
3	[Branch1]	11	[=N]
4	[=Branch1]	12	[=C]
5	[#Branch1]	13	[#C]
6	[Branch2]	14	[S]
7	[=Branch2]	15	[P]
All other symbols are assigned index 0			

Then, *N* symbols from 
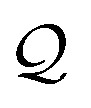
 (or all symbols in 
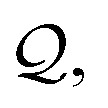
 if fewer exist) are consumed to form a new SELFIES string, and with start symbol 
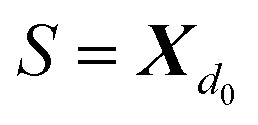
 (instead of *S* = *S* as before), this substring is recursively derived into a SMILES string *ρ*_0_. We take *ρ* = *ε* if *ρ*_0_ = *ε*, and *ρ* = (*ρ*_0_) otherwise.§§A minor technicality occurs if *ρ*_0_ starts with a branch parentheses (, in which case *ρ* is of the form ((*α*_1_),…,(*α*_*m*_)*α*_*m*+1_) for strings *α*_*k*_ ∈ Σ* that do not start with (. This would result in an invalid SMILES string because branches cannot start with other branches in SMILES. To amend this, we naturally interpret and replace *ρ* with the string (*α*_1_),…,(*α*_*m*_)(*α*_*m*+1_).

Examples: To provide an overview of branch derivation, we translate a SELFIES string representing acetic acid:20
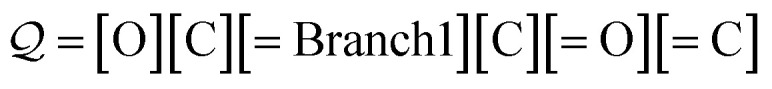


Processing the first two SELFIES symbols [O][C] results in the string OC *X*_3_, after which the symbol [=Branch1] is dequeued. Since *ℓ* = 1, we consume the next symbol [C] in 
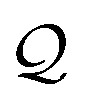
 and identify it with *N* = 1. Hence, we create the SELFIES substring [=O] from popping the next symbol in 
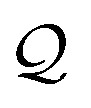
 and, with start symbol *X*_2_, recursively derive it into the SMILES substring *ρ* = (=O). Then, performing the replacement in eqn (18) gives the string OC(=O) *X*_1_, and processing the last symbol [=C] in 
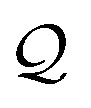
 finally produces a SMILES string OC(=O)C for acetic acid. Another SELFIES string that corresponds to OC(=O)C is:21
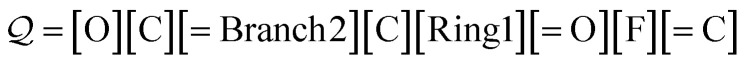


The derivation is largely similar to that before. The major difference is that when the branch symbol is dequeued, the next *ℓ* = 2 symbols [C][Ring1] are identified with *N* = 1 + 16(0) + 1 = 2, and then, the SELFIES substring [=O][F] is used to again derive *ρ* = (=O).

### 3.5. Ring derivation

The final feature that is necessary to capture the diverse variety of molecules is the ability to encode ring closures. In SMILES, this is achieved by paired numeric tags that indicate two separate atoms are joined together; for example, CC1CCC1 (methylcyclobutane). By adding bond characters before the numbers, SMILES can also specify ring closures of higher bond orders, such as C=1CCCC=1 (cyclopentene). In SELFIES, ring closures are specified by ring symbols, which behave similarly to branch symbols. The derivation process extends that in Section 3.4.

Per eqn (6), there are two forms of SELFIES ring symbols. To simplify the ensuing discussion, however, we will begin by only considering the first form. When a ring symbol [*β* Ring *ℓ*] is popped from the queue of SELFIES symbols 
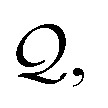
 a nonterminal symbol *A* in the current derived string is used to specify a production rule. If *A* = *S*, then we apply the rule *A* → *A*, and the ring symbol is effectively skipped. If *A* = *Xi*, then we replace:

22 ***A*** → ***X***_*i*−min(*i*,*d*(*β*))_

In addition, we consume the next *ℓ* symbols of 
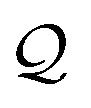
 (or all symbols in 
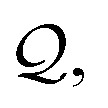
 if fewer exist) to specify a number 
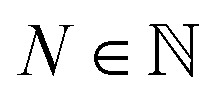
 by eqn (19). Then, the ring symbol would indicate that a ring closure should be formed between the ring-initiating atom and the *N*-th atom previously derived from it (or simply, the first atom if less than *N* such atoms exist). Here, the derivation order is the order in which atoms are realized through the production rules in eqn (10) and (11). By ring-initiating atom, we also mean the atom at which bonds would be made if the ring symbol were instead an atom symbol. Often, this coincides with the last-derived atom, as is the case in:

23 NC(C)COC*†***X***_4_

where the ring-initiating and last-derived atoms are marked with an asterisk and dagger, respectively. However, this is not the case when the last-derived atom lies within a fully-derived branch:

24 NC(C)COC*(C)(C†)***X***_1_

For brevity, we will refer to the ring-initiating atom as the right ring atom and its counterpart the left ring atom, as the latter precedes the former in a SMILES string under derivation order.

Although a ring symbol specifies a closure between the left and right ring atoms, such a bond cannot be naively added since it may cause valences to be violated for the left ring atom immediately (*e.g.*, consider the case where this atom has already attained its maximum valence) or in the future. Hence, SELFIES postpones the creation of ring closures to a final post-processing step. Instead, the ring closure candidates are pushed to a temporary queue 
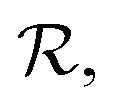
 and once all the SELFIES symbols have been processed, the items in 
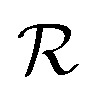
 are revisited in first-to-last order. Based on the state of the ring atoms, a candidate may be rejected (and no ring bond is made) or executed.

Specifically, given a potential ring closure indicated by symbol [*β* Ring *ℓ*], let *m*_1_ and *m*_2_ be the number of additional bonds that the left and right ring atoms can make, respectively. If *m*_1_ = 0 or *m*_2_ = 0, we must reject the candidate since adding the ring closure would exceed one of the valences of the ring atom. The candidate is also rejected if its left and right ring atoms are not distinct, to avoid unphysical self-loops. Otherwise, the candidate is accepted, and, assuming there is no pre-existing bond between its two ring atoms, we form a new bond of order *d*_0_ = min(*d*(*β*_1_), *m*_1_, *m*_2_) between them. If a prior bond does exist (*e.g.*, if a duplicate ring closure is specified earlier in 
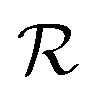
), then we increment the order of this existing bond as necessary. That is, if the existing bond is of order *d*_1_, then we promote it to a bond of potentially-higher order min(3, *d*_1_ + *d*_0_).

Examples: We translate a SELFIES string representing methylcyclobutane:25
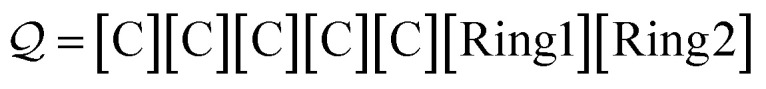


The first five symbols produce the string CCCCC ***X***_4_, after which the ring symbol [Ring1] is dequeued. Since *ℓ* = 1, the next and final symbol [Ring2] specifies a single ring bond between the final C and its *N* = 3rd preceding atom. This produces the SMILES string CC1CCC1. Note that incrementing the indexing symbol:26
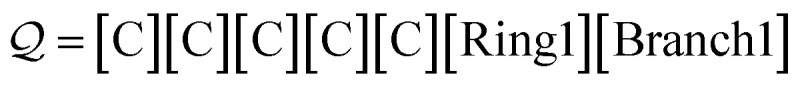


increments the distance of the ring closure, hence producing a SMILES string for cyclopentane C1CCCC1. Appending a copy of the ring and index symbols:27
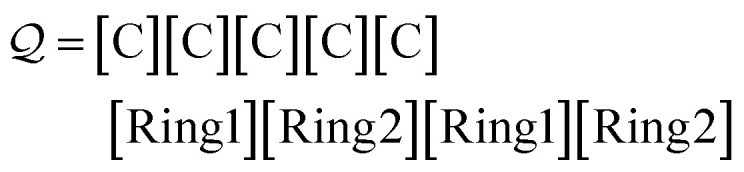
increments the bond order of the ring closure and produces the SMILES string CC=1CCC=1.

The second ring symbol form [*β*_1_*β*_2_ Ring *ℓ*] in eqn (3) behaves nearly identically to [Ring *ℓ*], and is used to support specification of stereochemistry across single ring bonds. The only difference occurs when a ring closure candidate produced by [*β*_1_*β*_2_ Ring *ℓ*] is accepted, and a new ring bond is added between the two ring atoms. In this case, if *β*_1_ ∈ {/, \}, then we add the bond character *β*_1_ before the numeric ring tag on the left ring atom, and similarly with *β*_2_ and the right ring atom. For instance, if the example eqn (25) used the symbol [/−Ring1] instead of [Ring1], then the derived SMILES string would be CC/1CCC1.

## 4. Library design

The selfies library is designed to be fast, lightweight, and user-friendly. A small but nice feature of selfies is that it also requires no extra dependencies. At its core, there are two functions that facilitate the interconversion between SELFIES strings and SMILES strings. For more advanced usage, we provide functions to customize the underlying semantic constraints that selfies enforces and operates upon. The default constraints are given in Table 4, and are intended for organic molecules with single, double, or triple bonds. Finally, we also provide a variety of utility functions for manipulating SELFIES strings. The following describes each type of function in more detail and provides potential use case examples. All code snippets are written in Python, with selfies being a Python library.

**Table 4:** The default constraints used by selfies. All atom types other than those explicitly listed below are constrained to 8 maximum bonds, which acts as a catch-all constraint

Element	Maximum bonds		
	Charge 0	Charge +1	Charge −1
H, F, Cl, Br, I	1	—	—
B	3	2	4
C	4	5	3
N	3	4	2
O	2	3	1
P	5	6	4
S	6	7	5

### 4.1. Core functions

SELFIES strings can conveniently be created from and turned into SMILES strings using the functions encoder( ) and decoder( ), respectively. The latter derives a SMILES string from a SELFIES string, using the procedure described in Section 3. The former performs the translation in the reverse direction such that passing a SMILES string through the composition decoder(encoder( )) is always guaranteed to recover a SMILES string that represents the same molecule (but not necessarily the original SMILES string itself). The recovered SMILES string will also maintain the molecular traversal order (*i.e.*, the specification order of the atoms) of the original string. The following excerpt defines a toy function roundtrip( ) that illustrates this:
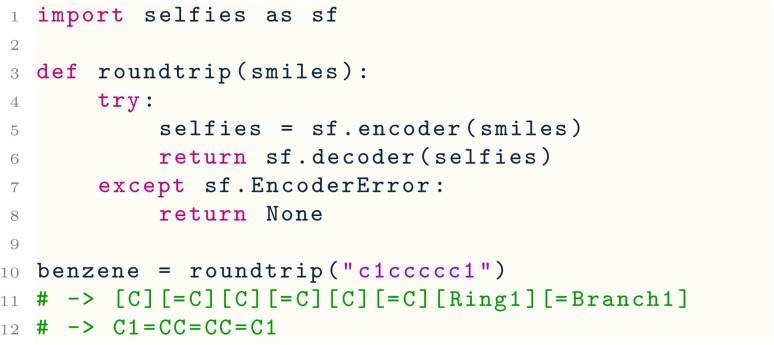


Line 5 translates the SMILES string for benzene into the SELFIES string in Line 11. Notably, SELFIES does not support aromatic atom symbols (*e.g.*, c) in the same way as SMILES, so encoder( ) performs an internal kekulization if it is passed an aromatic SMILES string. Line 7 guards against errors raised by encoder( ) when being passed SMILES strings that are syntactically invalid, semantically invalid (*i.e.*, violate the constraints described in the next subsection), or unsupported. An unsupported SMILES string uses features of SMILES that are not implemented in SELFIES, such as the wildcard * and quadruple bond $ symbols; the API reference of selfies further details which SMILES strings are currently supported. Line 10 applies the roundtrip( ) function to a SMILES string c1ccccc1 for benzene. Indeed, this round-trip translation recovers a SMILES string C1=CC=CC=C1 that is different than the original string, but still specifies the (kekulized) benzene molecule.

In greater detail, given an input SMILES string, encoder( ) first performs a kekulization if it contains any aromatic atom symbols, as was in the example above. Next, the actual translation process begins. In the simplest case, if the input represents a simple atom chain, then a translation to SELFIES is performed by essentially grouping each atom symbol with its preceding bond symbol, if any. For example, the SMILES string O=[13CH]C#N would be partitioned into O, = [13CH], C, #N and turned respectively into SELFIES symbols [O][=13CH1][C][#N]. Branches are recursively translated and the result is used to work backwards to find the appropriate branch and indexing symbols to prepend. If there are multiple plausible choices, we use the one in which the branch symbol [*β*Branch*ℓ*] has *ℓ* minimized and *β* representing the bond connecting the branch to the parent chain. For instance, C(=O)O is encoded as [C][=Branch1][C][=O][O] instead of [C][#Branch2][C][C][=O][O], despite both SELFIES strings producing C(=O)O under the derivation process. Finally, ring closures are handled similarly in that we work backwards to find the appropriate ring and indexing symbols. If there are multiple choices, we use the one in which the ring symbol [*β* Ring *ℓ*] (or [*β*_1_*β*_2_ Ring *ℓ*]) has *ℓ* minimized and *β* (or *β*_1_, *β*_2_) representing the bond of the ring closure.

#### 4.1.1. SELFIES and SMILES

The core functions of selfies interconvert between SELFIES and SMILES; and in Section 3, we present the method of interpreting SELFIES strings by deriving SMILES strings under a simple augmented grammar, following the previous SELFIES paper.^10^ However, it is important to note that SELFIES is not conceptually reliant on SMILES, and we may just as naturally interpret SELFIES strings through deriving molecular graphs. In fact, before version 2.0.0, both encoder( ) and decoder( ) were implemented as direct string-to-string translations. We have since refactored the functions to convert the input string to an intermediate graph-based representation, which is subsequently transcribed in the target representation. Future work could then expose this graph representation with a clean interface, allowing users to use selfies in a SMILES-independent manner.

#### 4.1.2. Random SELFIES

Since every string of SELFIES symbols can be derived into a valid SMILES string, we can generate random but valid SMILES strings by passing random SELFIES strings through decoder( ). To sample these SELFIES strings, we use the get_semantic_robust_alphabet( ) utility function, which returns a subset of semantically constrained SELFIES symbols:
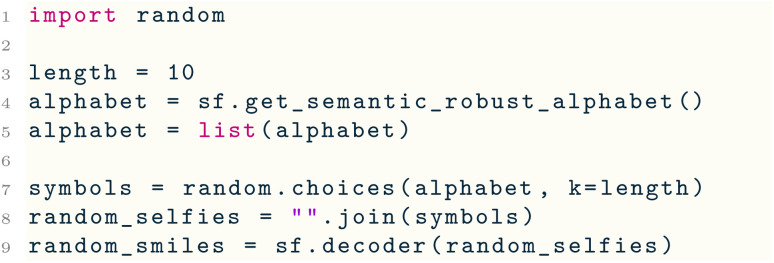


Note that by changing the pool of SELFIES symbols from which we sample from, we can change the distribution of produced molecules.

### 4.2. Explaining translation

To explain translations between SELFIES and SMILES, both encoder( ) and decoder( ) support an attribute flag that enables attributions of the output string symbol(s) to symbol(s) in the input string:
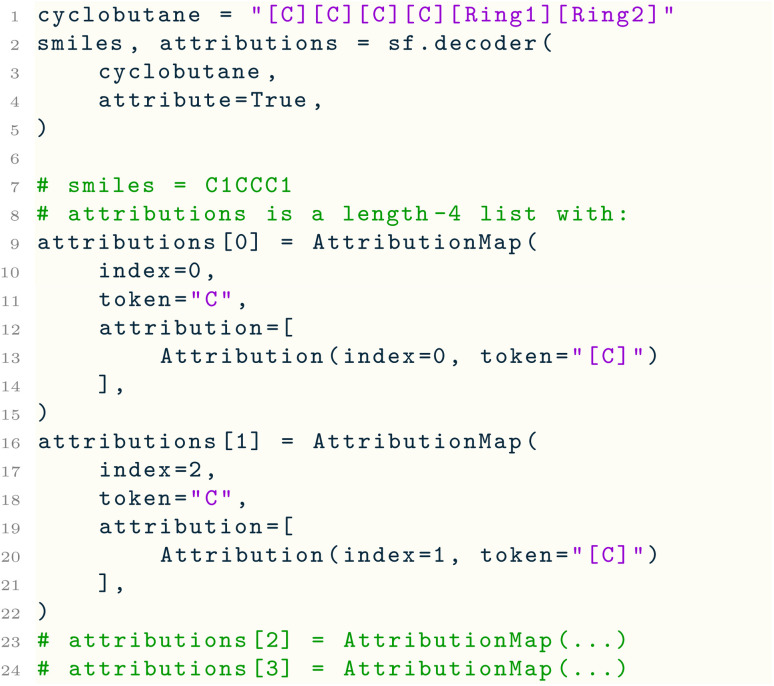


The attributions are a list of AttributionMap objects, one for each output symbol. Each AttributionMap contains the output symbol, its index, and a list of Attribution objects, each of which holds an input symbol (and its index) that is responsible for the output symbol. Note that a single output symbol may be attributed to multiple input symbols because it may be determined by both atom symbols and branch or ring symbols. Tracing the relationship between symbols can enable alignment between SMILES and SELFIES so that per-atom properties can be connected on both sides of the translation.

### 4.3. Customization functions

The selfies library dynamically constructs its derivation rules from a set of prespecified constraints, which dictate the maximum number of bonds that each atom type in a molecule may form. The derivation rules then ensure that each SELFIES string corresponds to a molecular graph satisfying the set constraints. By choosing a set of constraints in accordance with chemical valences, 100% robustness can be achieved. Specifically, selfies uses the constraints in Table 4 by default.

However, a limitation of the default constraints is that SELFIES cannot represent existing molecules that violate them, such as perchloric acid (which features a hypervalent Cl making 7 bonds). Moreover, the catch-all constraint may be too relaxed to ensure the validity of SELFIES strings containing atom types outside those in Table 4 (*e.g.*, Si, Se). Hence, users may wish to instead use custom constraints that are tailored to the SELFIES strings being worked with. To this end, selfies provides the key function set_semantic_constraints( ). The following provides a minimal example:
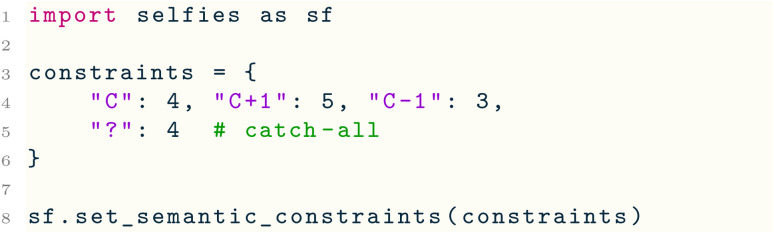


Here, the constraints dictionary encodes a set of custom constraints; specifically, explicit constraints on the neutral and ±1 charged variants of C (as in Table 4) and a catch-all constraint (of 4 maximum bonds). Line 8 then sets constraints as the underlying semantic constraints that selfies will operate under, which changes the subsequent behaviour of encoder( ) and decoder( ) appropriately. Note that the pre-existing constraints are fully replaced in Line 8; any constraint that is not explicitly specified in constraints would be thus removed.

For convenience, selfies provides a couple of preset constraints to serve as templates that can be easily modified. These can be obtained as follows:
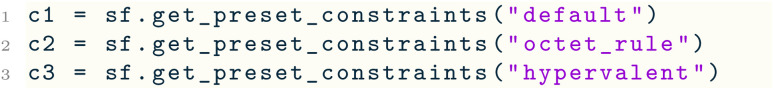


The currently-set constraints can also be viewed by:
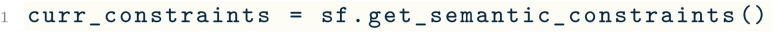


### 4.4. Utility functions

The selfies library provides a number of utility and convenience functions. Two basic utility functions are len_selfies( ), which computes the number of symbols in a SELFIES string, and split_selfies( ), which tokenizes a SELFIES string into an iterable of its constituent symbols:
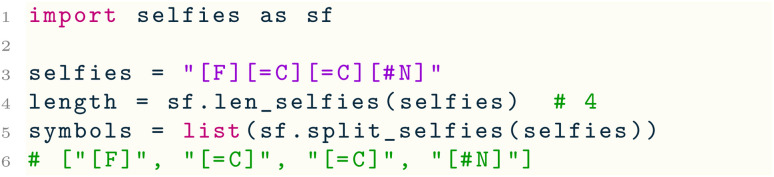


Furthermore, selfies includes functions to extract a vocabulary of symbols from a dataset of SELFIES strings, and to convert SELFIES strings into label or one-hot encodings. Consider the following example:
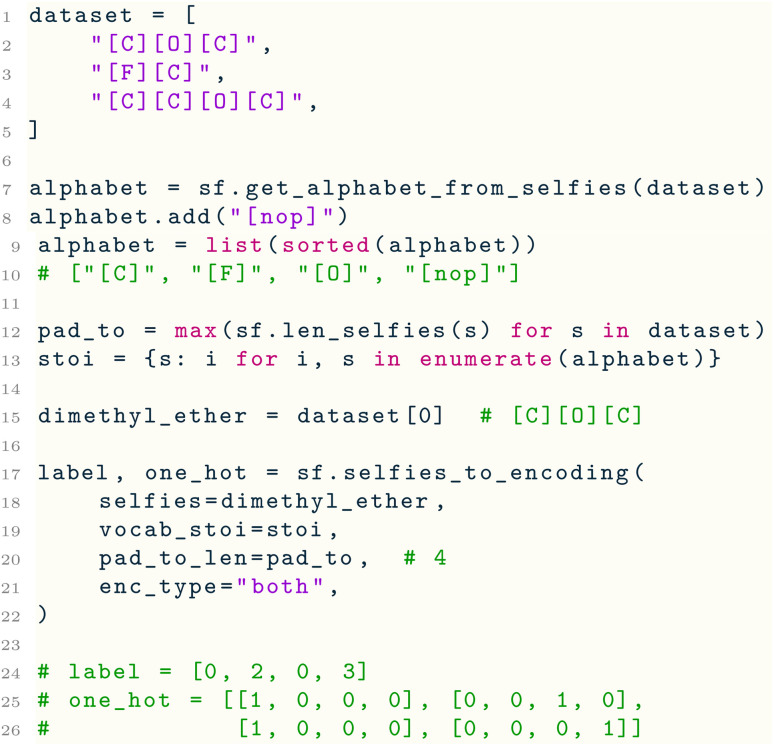


Here, we are given a list dataset of SELFIES strings. Line 7 uses a utility function of selfies to extract the set alphabet of SELFIES symbols that appear in the dataset, which is used in Line 13 to create a symbol to index mapping termed stoi. Next, lines 17–22 use another utility function selfies_to_encoding( ) to create a label and one-hot encoding of the first SELFIES string in the dataset. Under the hood, this function first pads the input string to length pad_to_len by appending to it sufficiently many copies of the symbol [nop] (for “no-operation”), which is a special padding symbol in selfies that is automatically ignored by decoder( ). Then, the padded SELFIES string is tokenized, and stoi is used to convert each of its symbols into integer labels and one-hot vectors. Since the padded SELFIES string may now contain [nop], this symbol must be added to stoi, which is done through Line 8. Lastly, the reverse encoding can be performed using the encoding_to_selfies( ) utility:
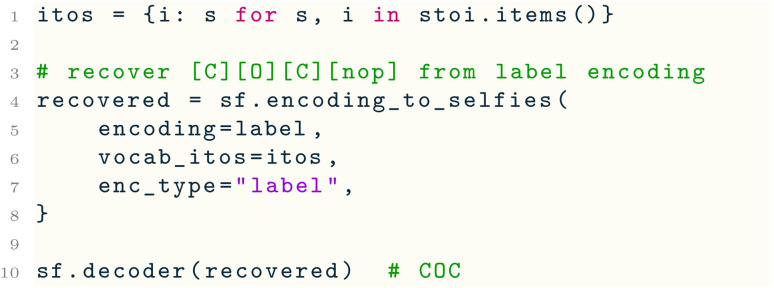


Table 5 summarizes the various utility functions introduced within this section.

**Table 5:** An overview of selfies utility functions

Function	Description
len_selfies( )	Computes the symbol length of a SELFIES string
split_selfies( )	Tokenizes a SELFIES string into its constituent symbols
get_alphabet_from_selfies( )	Extracts a minimal vocabulary from a dataset of SELFIES strings
selfies_to_encoding( )	Converts a SELFIES string into a label and/or one-hot encoding
encoding_to_selfies( )	Recovers a SELFIES string from its label and/or one-hot encoding
get_semantic_robust_alphabet( )	Provides an alphabet of semantically-constrained SELFIES symbols

## 5. Results and discussion

The selfies library is quick and efficient in its translation, despite being implemented in pure Python. To demonstrate this, we provide some simple benchmarks of its core functions encoder( ) and decoder( ). The following experiments were run on Google Colaboratory, which uses two 2.20 GHz Intel(R) Xeon(R) CPUs.

### 5.1. Roundtrip translation

Here, we consider the roundtrip translation task, where a SMILES string is translated to SELFIES and then back to SMILES (see Section 4.1). Specifically, we translate the Developmental Therapeutics Program (DTP) open compound collection,^22,23^ which contains a little over 300 k SMILES strings and is a set of molecules which have been tested experimentally for potential treatment against cancer and the acquired immunodeficiency syndrome (AIDS).^24^ Translating the full dataset into SELFIES strings with encoder( ) takes 136 s, and recovering the SMILES dataset using decoder( ) takes 116 s, for a total roundtrip translation time of 252 s. Fig. 2 plots how this roundtrip time scales with molecular size. Notably, we obtain all of these times by averaging over 3 replicate trials.

### 5.2. Random SELFIES

**Fig. 1:**
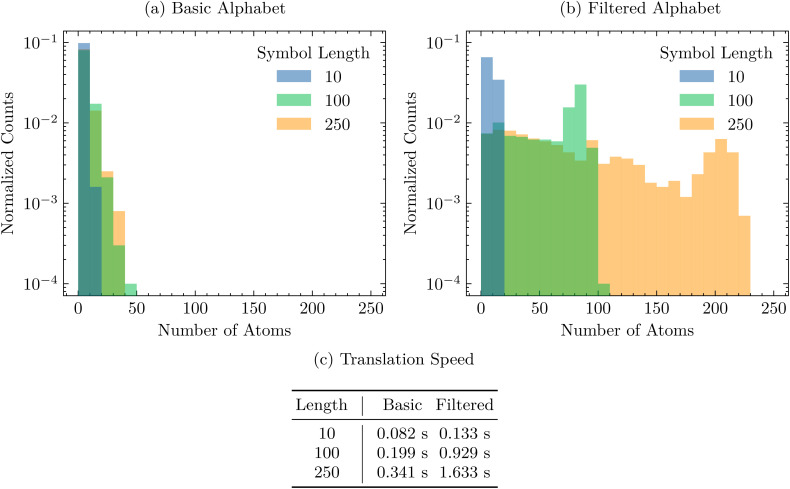
For a fixed alphabet 
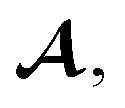
 1000 SELFIES strings were generated by uniformly sampling *L* symbols from an alphabet. Then, we plot the size distribution of the resulting molecules for varying symbol lengths *L*. (a) We take 
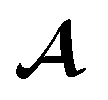
 to be the 69 symbols returned by get_semantic_robust_alphabet( ) under the default semantic constraints. (b) We filter the alphabet in (a) to 19 symbols by removing all atom symbols [*βα*] where *β* ∈ {=, #} or *ν*(type(*α*)) = 1, and removing all branch and ring symbols except for [Branch1] and [Ring1]. This decreases the chance that the SELFIES derivation process is terminated early, causing the derived molecules to be larger. (c) The time taken to translate each batch of random SELFIES strings to SMILES using decoder( ), measured by averaging over 20 replicate trials.

First, we sample 1000 fixed-length SELFIES strings and translate them to SMILES, per Section 4.1. We try this experiment with different symbol lengths and alphabets from which the SELFIES strings are built. Fig. 1 shows the resulting distribution of SMILES strings and the time it takes to decode each full batch of random SELFIES strings. Performing this experiment reaffirms the robustness of SELFIES and demonstrates the ease in which we can create random valid molecules without applying any filters, pre- or post-selection. In Fig. 1a, we show how SELFIES strings sampled from a basic alphabet translate to random molecules; an important observation is that the generated molecules are rather small, independent of the SELFIES length chosen. That is mainly caused by the inclusion of multi-bonds and low-valence atoms in the considered alphabet, which exhaust the available valences of the constituent atoms and then lead to an earlier termination of the derivation. A simple workaround is to instead use an alphabet without multi-bonds and low-valence atom types, as illustrated in Fig. 1b. Here, the molecular size distribution is shifted significantly towards larger molecules, especially when longer SELFIES string are sampled. Hence, this showcases how to create very large and valid random molecules.

**Fig. 2:**
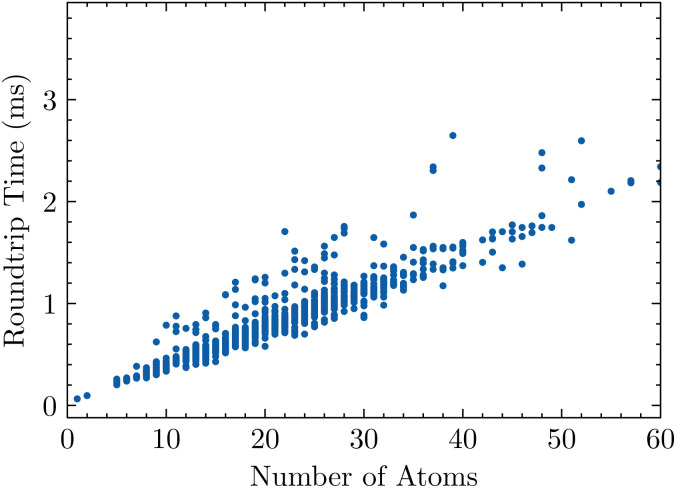
The roundtrip translation time of 1000 randomly-sampled SMILES strings from the DTP open compound collection as a function of size, measured in number of atoms.

## 6. Conclusions and outlook

Since its first release in 2019, the selfies library has undergone significant changes and experienced a drastic transformation in terms of both capabilities and code design. All of these modifications were executed with two major premises, namely, (1) extending its functionality and capability to support all features of the SMILES representation and (2) retaining or even improving upon its simplicity and user-friendliness. To achieve that, we implemented all necessary functionality in the library itself so that it does not require any other packages. Additionally, we added several utility functions to the library to support common use cases. Apart from these two prime goals, we also made significant efforts to make the implementation faster as SELFIES has been employed in many performance-critical applications and workflows.

Overall, the SELFIES community has grown rapidly and we are actively engaging in constructive discussions about the current implementation and future improvements. While selfies 2.1.1 supports almost all important features of SMILES, there are still many new features on our agenda. We outlined many of them in a recent perspective,^20^ for example, extensions to polymers, crystals, molecules with non-covalent bonds, or reactions. Our vision is that SELFIES will become a standard computer representation for molecular matter. We encourage the community to implement it into their workflows, report errors in the current implementation, and propose changes and new features that will help them to succeed in their goals.

## Data availability

The selfies library is available at GitHub (https://github.com/aspuru-guzik-group/selfies). Our benchmarking scripts were run on Google Colab and are also available at our repository (https://github.com/aspuru-guzik-group/selfies/blob/f38eeea4c8b60ce412fa917adb9258b89d4e8efc/examples/benchmark_v2_1_1.ipynb).

## Author contributions

A. L.: conceptualization (equal), data curation (lead), formal analysis (lead), investigation (lead), methodology (lead), software (lead), validation (lead), visualization (lead), writing – original draft (lead), writing – review & editing (lead). R. P.: conceptualization (equal), funding acquisition (supporting), methodology (equal), project administration (equal), software (equal), supervision (equal), writing – original draft (equal), writing – review & editing (equal). A. K. N.: conceptualization (equal), funding acquisition (supporting), methodology (supporting), software (supporting), writing – original draft (supporting), writing – review & editing (equal). A. D. W.: methodology (supporting), software (supporting), writing – original draft (supporting), writing – review & editing (supporting). M. K.: conceptualization (equal), methodology (equal), project administration (equal), software (equal), supervision (equal), writing – original draft (equal), writing – review & editing (equal). A. A. -G: conceptualization (equal), funding acquisition (lead), project administration (supporting), resources (lead), supervision (supporting), writing – review & editing (supporting).

## Conflicts of interest

There are no conflicts to declare.

## Supplementary Material
